# Strategies to improve patient retention on antiretroviral therapy in sub-Saharan Africa

**DOI:** 10.1111/j.1365-3156.2010.02506.x

**Published:** 2010-06

**Authors:** Anthony D Harries, Rony Zachariah, Stephen D Lawn, Sydney Rosen

**Affiliations:** 1 International Union against Tuberculosis and Lung DiseaseParis, France; 2 Department of Infectious and Tropical Diseases, London School of Hygiene and Tropical MedicineLondon, UK; 3 Medecins sans Frontieres, Medical DepartmentBrussels, Belgium; 4 Desmond Tutu HIV Centre, University of Cape TownCape Town, South Africa; 5 Center for International Health and Development, Boston UniversityBoston, MA, USA; 6 Health Economics and Epidemiology Research Office, Wits Health ConsortiumJohannesburg, South Africa

**Keywords:** HIV/AIDS, antiretroviral therapy, retention, treatment outcomes, lost to follow-up, Africa

## Abstract

The scale-up of antiretroviral therapy (ART) has been one of the success stories of sub-Saharan Africa, where coverage has increased from about 2% in 2003 to more than 40% 5 years later. However, tempering this success is a growing concern about patient retention (the proportion of patients who are alive and remaining on ART in the health system). Based on the personal experience of the authors, 10 key interventions are presented and discussed that might help to improve patient retention. These are (1) the need for simple and standardized monitoring systems to track what is happening, (2) reliable ascertainment of true outcomes of patients lost to follow-up, (3) implementation of measures to reduce early mortality in patients both before and during ART, (4) ensuring uninterrupted drug supplies, (5) consideration of simple, non-toxic ART regimens, (6) decentralization of ART care to health centres and the community, (7) a reduction in indirect costs for patients particularly in relation to transport to and from clinics, (8) strengthening links within and between health services and the community, (9) the use of ART clinics to deliver other beneficial patient or family-orientated packages of care such as insecticide-treated bed nets, and (10) innovative (thinking ‘out of the box’) interventions. High levels of retention on ART are vital for individual patients, for credibility of programmes and for on-going resource and financial support.

## Introduction

The massive and unprecedented scale-up of antiretroviral therapy (ART) in low and middle income countries in the last 5 years is probably the most outstanding achievement in the global fight against HIV/AIDS, where estimated numbers started on therapy have risen from 400 000 in 2004 to 4 million by the end of 2008 (WHO *et al.* 2009) In sub-Saharan Africa alone, 2.9 million patients were estimated to be receiving ART by December 2008, compared with about 25 000 in 2002. Tempering this success, however, is a growing concern about patient retention (patients who are alive and remaining on ART in the health system). A systematic review of ART programmes in sub-Saharan Africa in 2007 indicated that only 60% of patients were retained on therapy 2 years after starting ART, with deaths and losses to follow-up being the major causes of attrition (Rosen *et al.* 2007). In the real world of resource-constrained public health agencies and facilities, what can be done? Based on our personal experience with ART scale-up in Africa in government and mission health care facilities combined with research on ART programmes, we present 10 practical interventions that we believe can improve patient retention.

## 1. Set up and maintain simple, standardized monitoring systems

Every facility that provides ART must set up a simple, standardized monitoring system to track the numbers of patients starting therapy every month or every quarter and to determine at the end of every quarter five key standardized outcomes – those who are alive and on therapy, those who are dead, those who are known to have stopped treatment, those who have transferred out to another facility, and those who have been lost to follow-up or ‘defaulted’. Being alive and on therapy implies retention in care. Formal transfer outs from one facility to another are common as the number of facilities expands and patients seek treatment closer to home. Patients who transfer from one facility to another are still considered as being retained on ART in the health system, but only if they can be tracked to their new facility through linked record-keeping systems.

Reliable and regular reports of retention and attrition are carried out every 3 months, for example, in the resource-poor country of Malawi, where each facility performs its own quarterly and cumulative cohort outcome analysis with results checked and collated as a result of quarterly supervision and monitoring visits from Ministry of Health personnel and partners (Libamba *et al.* 2006). Treatment outcomes and their dates (or nearest month in which the outcome occurred) are rigorously recorded. This is labour intensive, and medical officers in charge of facilities have to allow staff sufficient time to complete the record-keeping and analysis. Good quality work should be rewarded, e.g. in Malawi, quarterly certificates of excellence are awarded to ART clinics for good record keeping of treatment cards and registers and accurate quarterly and cumulative outcome reports.

Unfortunately, and although carried out with the best of intentions, there is a tendency for donor-supported programmes to demand large amounts of data that relate to demographic and clinical features, adverse events, and biochemical and immunological tests. The predictable results in resource-constrained settings are poorly completed forms, incomplete data sets, and unreliable data on what counts, namely retention on therapy and attrition. ART programme designers and managers should resist this pressure, as timely collection of reliable data on the five standardized outcomes is hard enough to achieve on its own without over-loading the often manual monitoring system with a host of other parameters. This simple approach is vindicated by recent data from the Development of Antiretroviral Therapy in Africa (DART) trial in Uganda and Zimbabwe showing that treatment outcomes in the short to medium term are as good with simple clinical monitoring compared with clinical and laboratory monitoring (DART Trial Team 2010).

Electronic medical record systems can play an important role, particularly as numbers of patients and treatment sites increase. Experience from a wide range of sites across Africa has shown that for this to work properly, however, adequate human resources and staff training are essential (Forster *et al.* 2008). Moreover, appropriate, standardized soft-ware packages need to be developed to facilitate the use and expansion of this resource and to avoid the scenario of having as many different electronic information systems in use as there are administrative districts, NGO partners, or funding agencies.

## 2. Reliably ascertain true treatment outcomes

Good ART clinic practices must include reliable ascertainment of outcomes of death, stopped treatment and loss to follow-up (attrition parameters), and the formal recording of transfer outs from one ART facility to another. Death may be ascertained pro-actively by a relative or friend providing information to the clinic, or it may be discovered as part of active tracing of patients who fail to attend their clinic appointment. In some countries, national death registers can also be used, if available. Similarly, patients who have stopped therapy may inform the clinic, but it is more likely that this information will be discovered through active tracing. Patient transfers need to be structurally managed, through formal recording, otherwise these patients may also be recorded as lost to follow-up. Thus, the ascertainment of true outcomes is a crucial programmatic activity.

In Malawi, as in many other African countries, patients are classified as lost to follow-up if they fail to attend clinic or obtain medications for 3 months or more. In an operational research study in Northern Malawi to determine the true outcomes of these patients, 50% had died, 15% had stopped therapy, and 8% had transferred to another clinic and failed to inform their original clinic of this move (Yu *et al.* 2007). The results of this study were confirmed in a recent systematic review and meta-analysis (16 studies from sub-Saharan Africa), showing that 20% to 60% of patients who had been recorded as lost to follow-up had died (Brinkhof *et al.* 2009).

Patients who are lost to follow-up can be identified through regular scrutiny of paper-based treatment cards or use of electronic data-base systems. Pharmacy-based records of medication collection by patients can also be used to monitor patient retention (Nachega *et al.* 2006; Wood *et al.* 2008). Electronic pharmacy records in particular may provide an efficient means of generating lists of patients who have failed to pick up medication and who can then potentially be traced in the community. For example, iDART is a computerized software that requires no licence and is freely available to download at URL http://www.cell-life.org/idart/download/. This system allows easy management and follow-up of patients by pharmacists and programme management teams and has already been successfully used in seven large-scale ART programmes within four provinces in South Africa (Wood *et al.* 2008).

Clinics will vary in terms of their ability to trace patients who are either late for their appointment or who have been recorded as lost to follow-up. Well-resourced clinics that are not overly burdened with high patient loads may have outreach teams that can trace patients and reduce their loss to follow-up rate. Poorly resourced clinics cannot do this, but correct recording of addresses or the location of homes within villages or towns, using community networks, and tapping in to the expanding cell phone networks (Lester *et al.* 2006) should all be used to try and maintain patient contact. Where possible it is essential to conduct operational research to investigate and address the reasons for patient attrition, particularly deaths and loss to follow-up.

## 3. Reduce death rates

The ART-lower income country (ART-LINC) collaboration compared treatment outcomes from 18 ART programmes in lower-income settings (predominately in Africa) with those of 12 HIV cohort studies from Europe and North America and found that early mortality after initiation of ART was several times higher in resource-limited settings (Braitstein *et al.* 2006). From 8% to 26% of patients die in the first year of ART in African settings, with most deaths occurring in the first few months after starting treatment (Lawn *et al.* 2008). Risk factors for early deaths include low baseline CD4 lymphocyte count, initiation of therapy when already in WHO clinical stage 4, low body mass index and anaemia, with the main responsible diseases being tuberculosis (both diagnosed and undiagnosed), bacterial sepsis, cryptococcal meningitis and Kaposi’s sarcoma. Useful interventions to reduce this early mortality that should be implemented either before or simultaneously with initiation of ART include cotrimoxazole preventive therapy (Lowrance *et al.* 2007), active screening for tuberculosis among high-risk patients such as those with unexplained weight loss and/or unexplained chronic fever, and screening for those at high risk of cryptococcal meningitis with cryptococcal antigen testing and targeted pre-emptive treatment for those with positive results (Jarvis *et al.* 2009).

There seems little doubt that the cumulative risk of dying before and during ART is strongly associated with time spent at low CD4 counts (Lawn *et al.* 2009). At the end of 2009, WHO issued revised international ART guidelines for resource-limited settings recommending that all adults and adolescents with WHO Stage 3 and 4 disease and all those with a CD4 count < 350 cells/uL should receive ART (WHO 2009). However, for this to make much difference to patient management, there needs to be far better access to CD4 count testing, which will probably only happen when simple, inexpensive, point-of-care CD4 tests become available. The concept of ‘pre-ART’ care also needs to be championed. A package of regular care and support, which includes community awareness and empowerment, clinical assessment, CD4 count measurement and cotrimoxazole and isoniazid preventive therapy, for HIV-infected patients not yet eligible for ART would greatly assist in decreasing subsequent late presentation and high early mortality on ART (Lawn *et al.* 2010).

## 4. Ensure uninterrupted ART drug supplies

There must be no stock-outs of ART drug supplies, so that health care worker and patient confidence in the system is maintained. Accurate drug forecasting is dependent on data, and for those alive and on therapy, it is crucial to know what regimen (first line or second line) the patient is taking. The data recording exercise should be kept as simple as possible with those alive and on therapy stratified by type of regimen. ART programmes should prioritize a few standardized regimens that are offered at all ART facilities, with more sophisticated, tailor-made treatments being limited to centres of excellence. Electronic pharmacy-based systems would greatly assist in these endeavours. Linking the data system with drug forecasting and procurement was the approach adopted for national drug procurement during the first 4 years of ART scale up in Malawi, during which time there were no national or facility stock-outs (Harries *et al.* 2007). Secure drug supplies help to enhance credibility of the health facility and mitigate attrition from therapy.

## 5. Use simple, non-toxic, and free ART regimens

ART regimens need to be as non-toxic and simple to take as possible, and they must be offered free-of-charge to the patient. The most commonly used and least expensive ART regimen in resource-limited settings, especially in sub-Saharan Africa, is a fixed-dose drug combination of stavudine + lamivudine + nevirapine (Renaud-Thery *et al.* 2007). While not optimal in terms of tolerability or concurrent use with TB treatment, the ease of regimen administration allows the therapy to be given by paramedical staff, lay providers and expert patients in poorly staffed facilities. Stavudine is responsible for considerable late toxicity, but there are still limited alternatives in Africa. Recent research studies have demonstrated that a reduced dose of 30 mg twice daily for all patients is associated with good viral efficacy and a lower incidence of side effects (Ait-Mohand *et al.* 2008), and most African ART programmes now only use this lower dose. The WHO recommends the phasing out of stavudine and replacement with less toxic but more expensive alternatives, such as tenofovir (World Health Organization 2009), and if this can be carried out it should improve adherence.

Studies in Kenya (Zachariah *et al.* 2008) and Cameroon (Boyer *et al.* 2009), as well as cohort meta-analyses (Braitstein *et al.* 2006; Brinkhof *et al.* 2008), have all clearly shown that payment for ART drugs is associated with higher rates of death and loss to follow-up compared with drugs given free-of-charge. This evidence has led to policy change and a clear recommendation by the WHO that access to HIV/ART treatment at the point of service delivery should be free (Souteyrand *et al.* 2008).

## 6. Decentralize ART clinics and reduce frequency of visits for stable patients

ART clinics have to be brought nearer to patients’ homes, and thus ART care has to be decentralized to rural hospitals and health centres. This means task shifting, and the responsibility for the provision of ART care moving from doctors, to nurses, to paramedical officers and even to expert patients (Philips *et al.* 2008). Decentralization and task shifting has been shown to work in rural settings in both Malawi and Uganda (Jaffar *et al.* 2009; Massaquoi *et al.* 2009). The demand on patients to attend health facilities for drug pick-ups and clinical assessments must also be reduced. This can be achieved either by reducing follow-up visits to health facilities to once every 2 or 3 months or by providing home-based care through community nurses, expert patients or volunteers, home-based care being both feasible and cost-effective in rural settings (Jaffar *et al.* 2009). Another option that may be feasible in some countries is to allow non-medical facilities, such as schools or local government offices, to distribute pre-packaged drugs that have been labelled in advance by a central pharmacy.

## 7. Reduce indirect patient costs

Lifelong ART is demanding for patients, and attempts must be made to reduce and minimize indirect patient costs, for example, for transport or time spent out of the work place. Despite provision of free ART in poor South African townships, socioeconomic status within these communities remains a powerful predictor of risk of death or loss to follow-up (Cornell *et al.* 2009). Many patients have no income and have to choose, for example, between the providing food for their family or transport to the ART clinic. Similarly in Malawi, high transport costs have been associated with lower acceptance rates of ART among tuberculosis patients in rural communities and higher rates of loss to follow-up (Zachariah *et al.* 2006). Solutions include socio-economic interventions such as reimbursement for transport costs, decentralization of services within communities or home-based care, the latter being less expensive for patients compared with travel to clinics (Jaffar *et al.* 2009).

## 8. Strengthen ART links within and between the health service and the community

It is important to consider integration and streamlining of health services so that patients do not get lost within the general health system. For example, there was a high loss to follow-up of HIV-infected pregnant women who were referred to a community ART clinic in South Africa (Kaplan *et al.* 2008), part of the reason being difficulties experienced by the women in attending separate antenatal, ART and paediatric clinics. The same problem is confronted by HIV-infected tuberculosis patients, who often have to attend separate tuberculosis and ART clinics for monitoring and drug collection.

Every attempt must be made by ART clinics to link their services with the community, and particularly with associations of people living with HIV/AIDS. In rural Malawi, care packages such as home treatment of opportunistic infections, support to family carers, referral of patients with adverse drug reactions, adherence counselling and defaulter tracing have, not surprisingly, been associated with better ART treatment outcomes (Zachariah *et al.* 2007). In a large ART service within a poor South African township, a peer counsellor system has contributed to comparatively low rates of mortality and loss to follow-up (Lawn *et al.* 2006, 2007). These are all win–win solutions for patient retention in the face of the human resources crisis in Africa.

## 9. Use ART services to deliver other useful interventions

In Uganda, the provision of cotrimoxazole and insecticide-treated bed nets in addition to ART resulted in a considerable reduction in frequency of malaria attacks (Mermin *et al.* 2006). In Malawi, pilot studies demonstrated the feasibility of providing insecticide-treated bed nets through routine ART clinics, and this service was very popular with patients and health care providers (Makombe *et al.* 2007). There needs to be a degree of flexible thinking, followed by firm commitments, to use the ART programme as an opportunity to improve holistic linkages and provide additional services such as nutritional support and family planning, all of which may encourage better retention on treatment. Chronic, non-communicable diseases such as diabetes mellitus and hypertension are becoming important additional causes of morbidity and mortality in rural and urban settings in Africa (Tollman *et al.* 2008). The principles and practices of providing life long chronic structured care are being learnt from ART programmes (Harries *et al.* 2009), and it makes sense to provide integrated chronic care for infectious and non-infectious diseases together. Models already exist, for example a chronic care clinic in Cambodia that provided structured treatment for patients with HIV/AIDS, diabetes mellitus and hypertension (Janssens *et al.* 2007).

## 10. Innovative (thinking ‘out of the box’) interventions

It will be crucial as the burden of patients on ART grows to think out of the box about how to support life-long adherence to medication and compliance with follow-up. This could include interventions such as twice daily radio announcements reminding patients to take their medication; ART stations at work places; and pick-ups of pre-packaged ART from local general supply stores, schools, or community-based ART refill sites rather than always from the clinic. These new modes of delivery, which will inevitably involve loss of control from the medical profession, will need to be piloted and tested through operational research.

In conclusion, the initial enthusiasm for ART scale-up may be threatened if ART retention rates are poor or progressively deteriorate. The international donor community will want to see its money well spent, and it is therefore beholden on ART programmes to collect the necessary data and to use all measures possible to ensure high retention and adherence to therapy.
